# Supplementary material for: Investigating possibilities for surveillance of long term chlamydia complications in the Netherlands: A qualitative study
Source: PLoS One. 2024 Jun 11;19(6):e0305279. doi: 10.1371/journal.pone.0305279 (PMC11166304; doi:10.1371/journal.pone.0305279)
Supplement: S1 File — (PDF) [file pone.0305279.s001.pdf]

Semi-structured interview guide – surveillance of chlamydia related long term complications

Subject 1 – diagnosis

- How often do you see patients with STI’s in your consults? (per week/month/year).
- How often do you see patients with long term complications related to chlamydia? (per week/month/year).

Discus following questions per complication (PID, EP, TFI)

- Could you explain the process of diagnosing this complication?
- What information is needed for a diagnosis?
- Could you give an example of a situation where it was hard to diagnose the patient?
- Would anything change in the process of diagnosis of this complication if the chlamydia testing strategy would change? And, if yes, what would change?

Subject 2 – registration

Discus following questions per complication (PID, EP, TFI)

- Could you explain the registration process for this complication?
- Are registration practices standardized within your profession?
- Do you refer patients with this complication?
- How do you register referrals?

Subject 3 – importance of surveillance

- Could you describe the trend of chlamydia related long term complications in the Netherlands?
- Could you describe the trend of chlamydia related long term complications in your region and/or workplace?
- What would you expect the trend to be in the upcoming years, and why?
- What information sources do you use for following the trend?
- Do you think this trend is trustworthy, and why?
- Are you interested in following the trend? And, if yes, how would you like to be informed?
- Could you explain how you would use trend information in your work?

Subject 4 – opportunities for surveillance

- Could you explain how you think trends of chlamydia related long term complications should be monitored over time?
- What is the importance of monitoring trends of chlamydia related long term complications?
- Could you explain who should be giving and receiving the information on trends? And why?

Demographics

- Gender
- Age category (10 years)
