# Supplementary material for: Investigating possibilities for surveillance of long term chlamydia complications in the Netherlands: A qualitative study
Source: PLoS One. 2024 Jun 11;19(6):e0305279. doi: 10.1371/journal.pone.0305279 (PMC11166304; doi:10.1371/journal.pone.0305279)
Supplement: Attachment — Submitted filename: Response to Reviewers.docx [file pone.0305279.s003.docx]

Thank you for giving us the opportunity to resubmit our manuscript entitled “*Investigating possibilities or surveillance of long term chlamydia complications in the Netherlands: a qualitative study*”. Please find our response to the editorial and reviewer comments below.

We look forward to hearing from you.

On behalf of all authors,

Elke den Boogert

Response to editorial comments:

**Please review your reference list to ensure that it is complete and correct. If you have cited papers that have been retracted, please include the rationale for doing so in the manuscript text, or remove these references and replace them with relevant current references. Any changes to the reference list should be mentioned in the rebuttal letter that accompanies your revised manuscript. If you need to cite a retracted article, indicate the article’s retracted status in the References list and also include a citation and full reference for the retraction notice.**

Thank you for pointing this out. We double checked our reference list and we saw that reference 19 is in the reference list but not in the text. Reference 18 in line 349 should have been reference 19. We now changed it to reference 23 in line 349, as we also added four references earlier in the manuscript (line, 106-107, 109, 154 and 299).

Response to reviewer comments

**Reviewer #1: Thank you for responding to the reviewer questions. I think the manuscript has significantly improved and may be published after the points below have been addressed:**
**Methods section**
**1. Line 121 – Thank you for adding the below in response to my previous comment regarding which qualitative approach/research paradigm you have used: “An interpretive qualitative research approach was used for data collection by developing a semi-structured interview guide…”. Would you mind elaborating on this approach and providing a reference for this?**

Thank you. We updated the manuscript by including a section on study design in the
methods in line 105-111:
“*Design*

A qualitative interpretative phenomenological method was used (18). Phenomenology seeks to understand the individual interpretation and view on a phenomenon (18). In our study we were interested in individual interpretation of guidelines and registration. Diagnosis of complications is set in guidelines, however interpretation seems difficult (19). To better understand the experiences of professionals in applying these guidelines and related registrations in their daily practice, we conducted phenomenological interviews.”

Also, we added the approach to line 113 ‘This interpretive phenomenological qualitative study included …’

**Also, you have added this to the ‘data collection’ section, but since a research paradigm usually does not only relate to data collection, but also to data analysis and interpretation, it would be good if you could elaborate how your overall research paradigm influenced also data analysis and interpretation.**

Thank you. We added more information about the design to the methods, see our answer above. Also, we added to line 152-154 ‘To identify individual experience with interpreting guidelines and registrations in our qualitative dataset we conducted an interpretive thematic analysis using MAXQDA Plus 2022 (Release 22.0.0) for data management (20).’

**2. The authors have indicated that they have checked the “manuscript according to the SRQR checklist and added the checklist to this letter (A Word file named SRQR Checklist)” – However, the checklist has not been included in the pdf file and I have not been able to find the SRQR checklist elsewhere in the system for some reason…**
We added the SRQR checklist as ‘Other’ in the submission system and was available to access at the end of the pdf file. We updated the checklist according the last comments from the reviewer and also added it below this rebuttal letter as well as ‘Other’ material in the system.

**Discussion**
**3. Line 287 – First, thank you for partly addressing my previous question regarding sample size and saturation by making changes to the methods section and the discussion. This is now clearer, apart from line 287, where you say “Saturation for SHC doctors was reached after two interviews, a third interview was conducted to confirm saturation.” Instinctively, the reader might think that after only two interviews, there might still be a high chance of missing important themes or perspectives – although in a highly homogenous sample, such as SHC doctors, this might be less of an issue, if referring only to ‘main themes’, as you had done for GPs; there might also be sampling bias for the first few participants, especially if a ‘network/ snowball sampling’ approach has been used. I would therefore be careful with such statements, or justify them better with reference to the literature (including the literature I had referred to during my previous review).**

Thank you for this further explanation and comment. We changed the section in line 294-299. The section now reads: “Saturation for the main themes was reached after two interviews with SHC doctors, mainly since they explained that they hardly ever diagnose chlamydia related complications. A third interview was conducted to confirm their responses regarding the main themes. However, since network and snowball sampling could result in a more limited specificity, and thus requiring more participants to reach saturation, it might still be possible that other views among SHC doctors exist (21).

**Entire manuscript
4. I have spotted a number of grammar mistakes – for example:
- Abstract, line 33 – Instead of “the possibility to introducing” it should say “the possibility of introducing”
- Introduction, line 91 – Instead of “monitor trends in chlamydia.” it should say something like “monitor trends in chlamydia infections.”
- Line 96 – Instead of “More specific,, we aimed to understand...” it should read “More c, we aimed to understand…”
After the revision, the entire manuscript will therefore need to be cross-checked for typos/ grammar mistakes.**

Thank you. We changed the abstract and the introduction as suggested. We checked the entire manuscript and made additional small changes throughout the manuscript.
It is unclear to what mistake the reviewer is referring in line 96. We don’t see a grammar mistake.

**Reviewer #2: The reviewers have addressed all comments raised as part of the original review. I have just a few minor comments:

Line 81 - there is a word missing in this sentence
Line 84 - missing punctuation
Line 217-219 - this sentence is unclear; CAT should be defined on first use**

Thank you for your comments. We added the word ‘for’ in line 81, the sentence now reads: “In the Netherlands, guidelines recommend chlamydia testing for persons experiencing symptoms, their contacts, and risk groups regardless of symptoms such as persons who had unprotected sex (15).”

We added the punctuation in line 85.

CAT is defined in line 216, when it is used for the first time. For clarity, we also added the definition to line 231.

|  | **Standards for Reporting Qualitative Research (SRQR)*** |  |
| --- | --- | --- |
|  | <http://www.equator-network.org/reporting-guidelines/srqr/> |  |
|  |  | **Page/line no(s).** |
| **Title and abstract** | |  |
|  | **Title** - Concise description of the nature and topic of the study Identifying the study as qualitative or indicating the approach (e.g., ethnography, grounded theory) or data collection methods (e.g., interview, focus group) is recommended | Page 1 / line 1-2 |
|  | **Abstract** - Summary of key elements of the study using the abstract format of the intended publication; typically includes background, purpose, methods, results, and conclusions | Page 2 – line 25 - 48 |
|  |  |  |
| **Introduction** | |  |
|  | **Problem formulation** - Description and significance of the problem/phenomenon studied; review of relevant theory and empirical work; problem statement | Page 3 and 4 – line 50-94 |
|  | **Purpose or research questio**n - Purpose of the study and specific objectives or questions | Page 4 – line 90-94 |
|  |  |  |
| **Methods** | |  |
|  | **Qualitative approach and research paradigm** - Qualitative approach (e.g., ethnography, grounded theory, case study, phenomenology, narrative research) and guiding theory if appropriate; identifying the research paradigm (e.g., postpositivist, constructivist/ interpretivist) is also recommended; rationale** | Page 5 – line 105-110 and line 112 |
|  | **Researcher characteristics and reflexivity** - Researchers’ characteristics that may influence the research, including personal attributes, qualifications/experience, relationship with participants, assumptions, and/or presuppositions; potential or actual interaction between researchers’ characteristics and the research questions, approach, methods, results, and/or transferability | Page 6 – line 141 - 145 |
|  | **Context** - Setting/site and salient contextual factors; rationale** | Page 5 – line 116-121 |
|  | **Sampling strategy** - How and why research participants, documents, or events were selected; criteria for deciding when no further sampling was necessary (e.g., sampling saturation); rationale** | Page 5 – line 111-121 |
|  | **Ethical issues pertaining to human subjects** - Documentation of approval by an appropriate ethics review board and participant consent, or explanation for lack thereof; other confidentiality and data security issues | Page 6 – line 124-126 and Page 7 – line 155-158 |
|  | **Data collection methods** - Types of data collected; details of data collection procedures including (as appropriate) start and stop dates of data collection and analysis, iterative process, triangulation of sources/methods, and modification of procedures in response to evolving study findings; rationale** | Page 5 and 6 – line 122-145 |
|  | **Data collection instruments and technologies** - Description of instruments (e.g., interview guides, questionnaires) and devices (e.g., audio recorders) used for data collection; if/how the instrument(s) changed over the course of the study | Page 5 – line 108-110, and page 6 line 127-139 + S1 |
|  | **Units of study** - Number and relevant characteristics of participants, documents, or events included in the study; level of participation (could be reported in results) | Page 7 – line 160-165 |
|  | **Data processing** - Methods for processing data prior to and during analysis, including transcription, data entry, data management and security, verification of data integrity, data coding, and anonymization/de-identification of excerpts | Page 6 and 7 – line 146-154 |
|  | **Data analysis** - Process by which inferences, themes, etc., were identified and developed, including the researchers involved in data analysis; usually references a specific paradigm or approach; rationale** | Page 6 and 7 – line 146-154 |
|  | **Techniques to enhance trustworthiness** - Techniques to enhance trustworthiness and credibility of data analysis (e.g., member checking, audit trail, triangulation); rationale** | Page 6 and 7 – line 146-154 |
|  |  |  |
| **Results/findings** | |  |
|  | **Synthesis and interpretation** - Main findings (e.g., interpretations, inferences, and themes); might include development of a theory or model, or integration with prior research or theory | Page 7 - 12 |
|  | **Links to empirical data** - Evidence (e.g., quotes, field notes, text excerpts, photographs) to substantiate analytic findings | Quotes are used throughout page 7-12 |
|  |  |  |
| **Discussion** | |  |
|  | **Integration with prior work, implications, transferability, and contribution(s) to the field -** Short summary of main findings; explanation of how findings and conclusions connect to, support, elaborate on, or challenge conclusions of earlier scholarship; discussion of scope of application/generalizability; identification of unique contribution(s) to scholarship in a discipline or field | Page 12-15 |
|  | **Limitations** - Trustworthiness and limitations of findings | Page 12 and 13 – line 277 – 298 and  Page 15 – line 339-345 |
|  |  |  |
| **Other** | |  |
|  | **Conflicts of interest** - Potential sources of influence or perceived influence on study conduct and conclusions; how these were managed | PLOSONE does not require this in the manuscript file, it will be added via the submission system. |
|  | **Funding** - Sources of funding and other support; role of funders in data collection, interpretation, and reporting | PLOS ONE does not require this in the manuscript file, it will be added via the submission system. |
|  |  |  |
|  | *The authors created the SRQR by searching the literature to identify guidelines, reporting standards, and critical appraisal criteria for qualitative research; reviewing the reference lists of retrieved sources; and contacting experts to gain feedback. The SRQR aims to improve the transparency of all aspects of qualitative research by providing clear standards for reporting qualitative research. |  |
|  |  |  |
|  | **The rationale should briefly discuss the justification for choosing that theory, approach, method, or technique rather than other options available, the assumptions and limitations implicit in those choices, and how those choices influence study conclusions and transferability. As appropriate, the rationale for several items might be discussed together. |  |
|  |  |  |
|  | **Reference:** |  |
|  | O'Brien BC, Harris IB, Beckman TJ, Reed DA, Cook DA. **Standards for reporting qualitative research: a synthesis of recommendations.** *Academic Medicine*, Vol. 89, No. 9 / Sept 2014  DOI: 10.1097/ACM.0000000000000388 |  |
|  |  |  |
|  |  |  |
